# The Effects of *HP0044* and *HP1275* Knockout Mutations on the Structure and Function of Lipopolysaccharide in *Helicobacter pylori* Strain 26695

**DOI:** 10.3390/biomedicines10010145

**Published:** 2022-01-10

**Authors:** Ai-Ning Liu, Kai-Wen Teng, Yongyu Chew, Po-Chuan Wang, Tram Thi Hong Nguyen, Mou-Chieh Kao

**Affiliations:** 1 Institute of Molecular Medicine, National Tsing Hua University, Hsinchu 30013, Taiwan; iwpa820814@gmail.com (A.-N.L.); lin66556@gmail.com (K.-W.T.); jynn1992@gapp.nthu.edu.tw (Y.C.); hongtramhs@gmail.com (T.T.H.N.); 2 Department of Gastroenterology, Hsinchu MacKay Memorial Hospital, Hsinchu 30013, Taiwan; wbccac@gmail.com; 3 Department of Life Science, College of Life Science, National Tsing Hua University, Hsinchu 30013, Taiwan

**Keywords:** *Helicobacter pylori*, lipopolysaccharide, GDP-D-mannose dehydratase (GMD), phosphomannomutase (PMM), phosphoglucomutase (PGM), outer membrane vesicles, bacterial virulence

## Abstract

*Helicobacter pylori* infection is associated with several gastric diseases, including gastritis, peptic ulcer, gastric adenocarcinoma and mucosa-associated lymphatic tissue (MALT) lymphoma. Due to the prevalence and severeness of *H. pylori* infection, a thorough understanding of this pathogen is necessary. Lipopolysaccharide, one of the major virulence factors of *H. pylori*, can exert immunomodulating and immunostimulating functions on the host. In this study, the *HP0044* and *HP1275* genes were under investigation. These two genes potentially encode GDP-D-mannose dehydratase (GMD) and phosphomannomutase (PMM)/phosphoglucomutase (PGM), respectively, and are involved in the biosynthesis of fucose. *HP0044* and *HP1275* knockout mutants were generated; both mutants displayed a truncated LPS, suggesting that the encoded enzymes are not only involved in fucose production but are also important for LPS construction. In addition, these two gene knockout mutants exhibited retarded growth, increased surface hydrophobicity and autoaggregation as well as being more sensitive to the detergent SDS and the antibiotic novobiocin. Furthermore, the LPS-defective mutants also had significantly reduced bacterial infection, adhesion and internalization in the in vitro cell line model. Moreover, disruptions of the *HP0044* and *HP1275* genes in *H. pylori* altered protein sorting into outer membrane vesicles. The critical roles of *HP0044* and *HP1275* in LPS biosynthesis, bacterial fitness and pathogenesis make them attractive candidates for drug inventions against *H. pylori* infection.

## 1. Introduction

*Helicobacter pylori* (*H. pylori*) is a Gram-negative, microaerophilic spiral-shaped bacterium which infects more than half of the world’s population [1,2,3]. As a notorious human pathogen, *H. pylori* employs multiple virulence factors, such as CagA, VacA, UreA and lipopolysaccharide (LPS), for environmental adaptation and host colonization during the process of host–microbe interaction [4,5,6]. It has been considered as one of the major etiological factors for the development of gastritis, peptic ulcer, gastric cancer and mucosa-associated lymphoid tissue (MALT) lymphoma, and is ranked as a group one carcinogen by the World Health Organization (WHO) [7,8,9].

**Figure 1:**
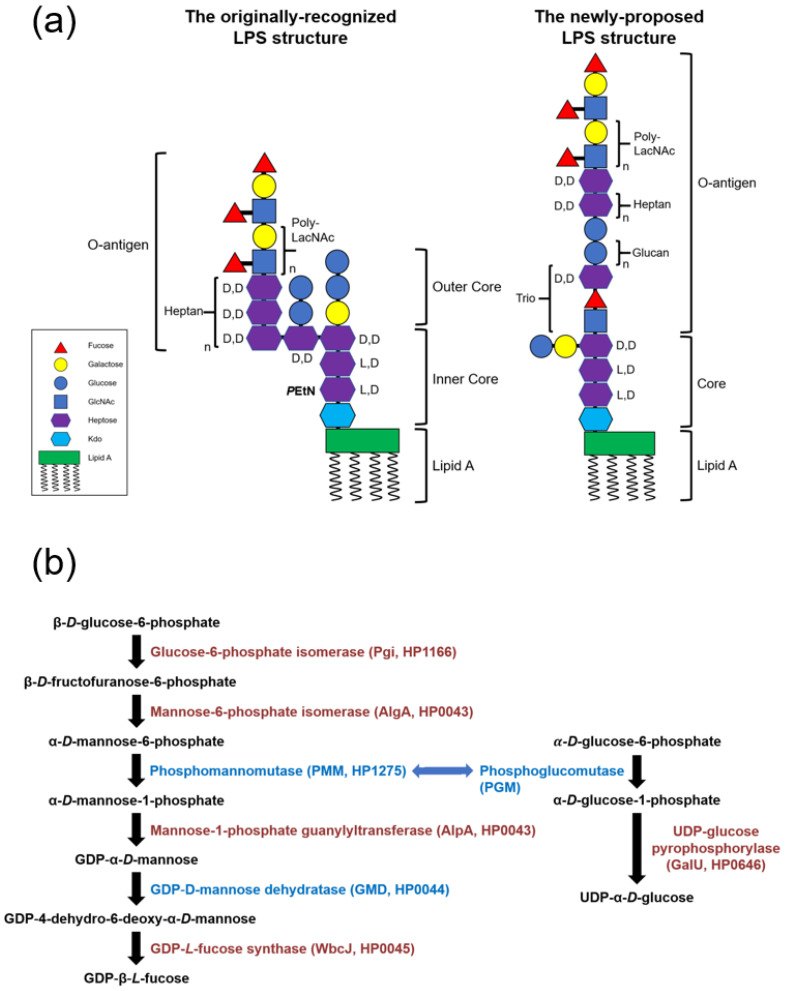
Diagrams of the LPS structure and fucose biosynthetic pathway in *H. pylori.* (**a**) The originally well-recognized LPS structure of *H. pylori* wild-type strain 26695 and the re-defined LPS structure in *H. pylori* wild-type strains G27 and 26695. (**b**) The putative fucose biosynthetic pathway in *H. pylori* wild-type strain 26695 (the enzymes tested in this study are marked in blue).

One of the major characteristics of Gram-negative bacteria is the presence of LPS on the bacterial outer membrane (OM) [10]. As a virulence factor, LPS is essential for the survival of most Gram-negative bacteria. Functionally, LPS maintains the integrity of the overall bacterial structure and protects the bacteria from erosion by several chemicals or anti-bacterial agents [11]. Furthermore, LPS on most Gram-negative bacteria can also induce strong inflammation responses during bacterial infection [12]. In *H. pylori*, a redefined LPS structure and several enzymes responsible for glycan transfer were revealed by recent research findings (Figure 1a) [13,14]. The complete LPS structure, known as smooth-form LPS (S-LPS), is composed of lipid A, a relatively shorter core oligosaccharide, and a linear O-antigen polysaccharide. The unique O-antigen structure of *H. pylori*, which is different from the previously well-recognized branching structure, contains a “Trio” region (heptose-fucose-*N*-acetylglucosamine (GlcNAc)), a heptan repeat unit, a glucan repeat unit and the Lewis X and Lewis Y antigens in the terminus.

Fucose is a building block of the Lewis X and Lewis Y antigens in the LPS O-antigen of *H. pylori* [15]. By the functional characterization, the Lewis antigens decorated on the *H. pylori* LPS were suggested to be involved in the bacterial adhesion and colonization, the regulation of host immune responses and the adaptation to the gastric environment through molecular mimicry [16,17,18,19]. In addition, fucose is not only decorated on the LPS terminus but also involved in the backbone of the LPS structure of *H. pylori*. A recent study showed that the deficiency of a fucosyltransferase, HP0102, in *H. pylori* wild-type strain G27 resulted in a truncated LPS which lost most of the O-antigen polysaccharide [14]. The GDP-L-fucose is generally synthesized by two routes: (1) a de novo synthetic pathway starting from GDP-α-D-mannose and (2) a salvage pathway starting from free L-fucopyranose [20]. The de novo biosynthetic pathway of fucose in many species, including *H. pylori*, has been characterized sporadically [21,22,23]. In *H. pylori* the biosynthetic process is suggested to begin with the conversion of β-D-glucose-6-phosphate into GDP-α-D-mannose, followed by the transformation of GDP-α-D-mannose into GDP-L-fucose. The complete biosynthetic pathway of fucose in *H. pylori* is shown in Figure 1b and described as follows: (1) the conversion of β-D-glucose-6-phosphate into β-D-fructofuranose-6-phosphate by the glucose-6-phosphate isomerase (Pgi, HP1166); (2) the formation of α-D-mannose-6-phosphate, catalyzed by the isomerase activity from the mannose-6-phosphate isomerase (AlgA, HP0043); (3) the conversion of α-D-mannose-6-phosphate into α-D-mannose-1-phosphate by the phosphomannomutase (PMM, HP1275); (4) the formation of GDP-α-D-mannose by the transferase activity from the mannose-1-phosphate guanylyltransferase (AlgA, HP0043); (5) the conversion of GDP-α-D-mannose into GDP-4-dehydro-6-deoxy-α-D-mannose by the GDP-D-mannose dehydratase (GMD, HP0044); and (6) the formation of GDP-L-fucose by the GDP-L-fucose synthase (WbcJ, HP0045).

GMD is an enzyme that catalyzes the elimination of water from GDP-α-D-mannose to form GDP-4-dehydro-6-deoxy-α-D-mannose, and belongs to the enzyme family of lyases. The enzymatic function, physical characteristics and structure of GMD have been identified in *Escherichia coli* (referred to as RfbD) and *Aneurinibacillus thermoaerophilus* [24,25,26]. The product of GMD, GDP-4-dehydro-6-deoxy-α-D-mannose, is essential for many bacteria since it serves as a key intermediate in several deoxyhexose biosynthetic pathways, including GDP-D-rhamnose, GDP-L-fucose, GDP-6-deoxy-D-talose and GDP-D-perosamine [27].

PMM is an enzyme that catalyzes the chemical interconversion between α-D-mannose-6-phosphate and α-D-mannose-1-phosphate, and belongs to the enzyme family of isomerases. The enzymatic function, physical characteristics and structure of PMM have been characterized in *Xanthomonas campestris* (referred to as XanA) and *Psedomonas aeruginosa* (referred to as AlgC) [28,29,30,31]. It is worth noting that the enzyme encoded by *P. aeruginosa algC* not only has the activity of PMM, but also has the activity of phosphoglucomutase (PGM, Figure 1b) [29]. PMM is a highly reversible phosphoryltransferase, and the disruption of PMM was shown to cause the formation of defective LPS. In *P. aeruginosa* the mutation of *algC* also resulted in the loss of PGM activity, and produced a truncated LPS with no detectable O-antigen [32].

Previous studies conducted in our laboratory have shown that a disruption in the LPS core oligosaccharide of *H. pylori* significantly reduced the efficiency of host colonization and thus the bacterial virulence [33,34,35]. Because fucose is also present in the center of the LPS structure of *H. pylori*, we expected that genetic defects of *HP0044* or *HP1275* would not only affect the biosynthesis of fucose but also alter the structure and function of LPS. In this study the *HP0044* and *HP1275* knockout mutants and their corresponding complementary mutants were constructed and the effects of the *HP0044* and *HP1275* mutations on the LPS profile, bacterial phenotype, fitness and virulence of *H. pylori* were examined. The results showed that the *HP0044-* and *HP1275*-disrupted mutations indeed caused LPS truncation. These two knockout mutants have retarded growth, higher surface hydrophobicity and autoaggregation as well as weaker ability of bacterial infection, adhesion and internalization compared to those in wild-type strain 26695. The protein profile and amount of CagA and VacA virulence factors in the outer membrane vesicles (OMVs) are also different between wild-type strain 26695 and the knockout mutants, thus suggesting that the sorting of proteins into OMVs is also affected by the *HP0044* and *HP1275* mutations. The current findings reveal the function and effect of GMD and PMM proteins in *H. pylori* in great detail and add a new understanding for future drug development against *H. pylori* infection.

## 2. Materials and Methods

### 2.1. Bacterial Strains, Plasmids and Growth Conditions

The bacterial strains and plasmids used in this study are listed in Table 1. The *H. pylori* wild-type strain 26695 (ATCC 700392; CagA^+^, VacA^+^) and the knockout mutants used in this study were grown on sheep blood (5% *w*/*v*) agar plates and incubated for 48 h to 72 h at 37 °C under microaerophilic conditions (5% O_2_, 10% CO_2_ and 85% N_2_). To change the growth conditions from solid to liquid media, bacteria were scraped off from agar plates and resuspended in Brucella broth media (BD Biosciences, Franklin Lakes, NJ, USA) with 10% fetal bovine serum (FBS, Biological Industries, Kibbutz Beit Haemek, Israel) and 1% IsoVitalex (Creative, Taipei, Taiwan). The flasks were then placed into an anaerobic chamber and cultivated for 48 h under a microaerophilic atmosphere at 37 °C with shaking at 140 r.p.m.

### 2.2. Construction of the HP0044 and HP1275 Knockout Mutants and the Corresponding Knockout Complementary Mutants

The *HP0044* or *HP1275* knockout mutants were constructed using a strategy based on gene splicing by overlap extension (SOEing) [36,37,38]. First, the upstream (582 base pairs (bps)) and downstream (583 bps) regions of the *HP0044* gene in addition to the upstream (679 bps) and downstream (796 bps) regions of the *HP1275* gene were amplified from the genomic DNA of *H. pylori* wild-type strain 26695 using primer pairs KO0044F1–KO0044R1/KO0044F2–KO0044R2 and KO1275F1–KO1275R1/KO1275F2–KO1275R2, respectively (Table 2). The two sets of generated polymerase chain reaction (PCR) products were then each joined via SOEing PCR with primer pairs KO0044F2/KO0044R1 or KO1275F2/KO1275R1 with a *BamHI* restriction site in the PCR products for subsequent insertion of the antibiotic resistance cassette to disrupt the *HP0044* and *HP1275* genes. The amplified 1165 bps fragment of *HP0044* and the amplified 1475 bps fragment of *HP1275* were purified and cloned into the pGEM-T vector (Promega, Madison, WI, USA) to generate the pGEM-T-HP0044 plasmid or the pGEM-T-HP1275 plasmid. Later, a chloramphenicol resistance cassette (Cm^r^) was inserted into the pGEM-T-HP0044 or the pGEM-T-HP1275 plasmid that had been digested with a *BamHI* restriction enzyme and treated with shrimp alkaline phosphatase (NEB, Ipswich, MA, USA). The resulting plasmid, pGEM-T-HP0044^d^::Cm^r^ or pGEM-T-HP1275^d^::Cm^r^, was confirmed by restriction digestion analysis, followed by natural transformation into *H. pylori* wild-type strain 26695.

To complement the aforementioned *HP0044* and *HP1275* knockout mutations, gene complementation was conducted by inserting the complementary genes into the locus of the *RdxA (HP0954)* gene. A 1561 bps fragment containing the promoter region of the *HP1563* gene (343 bps) and the open reading frame (ORF) of the *HP0044* gene (1173 bps) was PCR-amplified from *H. pylori* 26695 genomic DNA using the primer pairs Com0044F1–Com0044R1 and Com0044F2–Com0044R2, respectively (Table 2). Similarly, the 1719 bps fragment containing the promoter region of the *HP1563* gene (343 bps) and the ORF of the *HP1275* gene (1376 bps) was PCR-amplified from *H. pylori* wild-type strain 26695 genomic DNA using the primer pairs Com1275F1–Com1275R1 and Com1275F2–Com1275R2, respectively (Table 2). The two PCR products generated for each set were then joined via SOEing PCR with primers Com0044F1 and Com0044R2 for *HP0044*, or with primers Com1275F1 and Com1275R2 for *HP1275*. The resulting 1516 bps (for *HP0044*) and 1719 bps (for *HP1275*) products were then digested with *NcoI/KpnI* and ligated into the pGEM-T-RdxA_L_-T7_ter_-RdxA_R_ plasmid, which had been *NcoI/KpnI*-digested to create the pGEM-T-RdxA_L_-P*_HP1563_*-*HP0044*-T7_ter_-RdxA_R_ plasmid and pGEM-T-RdxA_L_-P*_HP1563_*-*HP1275*-T7_ter_-RdxA_R_ plasmid. The resulting complementary plasmids were then transformed into the *HP0044* and *HP1275* knockout mutants, respectively, by natural transformation.

### 2.3. SDS-PAGE and Immunoblotting Analysis

Protein samples were separated on 10% or 15% gels by sodium dodecyl sulfate–polyacrylamide gel electrophoresis (SDS–PAGE) and stained with 0.25% Coomassie brilliant blue R250 (Sigma-Aldrich, Burlington, MA, USA) or transferred to 0.45 μm nitrocellulose membranes (Millipore, Burlington, MA, USA) for immunoblotting analysis. The transferred membranes were blocked with 5% skim milk in phosphate-buffered saline (PBS) at room temperature for 1 h. Then, membranes were incubated at 4 °C overnight with one of the following primary antibodies: mouse anti-Lewis X monoclonal antibody (1:1000, ab3558, Abcam Biotechnology, Cambridge, UK), mouse anti-Lewis Y monoclonal antibody (1:1000, ab3359, Abcam Biotechnology), rabbit anti-CagA monoclonal antibody (1:2000, sc-25766, Santa Cruz Biotechnology, Dallas, TX, USA), rabbit anti-VacA monoclonal antibody (1:3000, HHP-5013-9, Austral Biologicals, San Ramon, CA, USA) and goat anti-UreA monoclonal antibody (1:3000, sc-21016, Santa Cruz Biotechnology). After washing the membranes were incubated with the following secondary antibodies: IRDye 800 CW goat anti-rabbit antibody (1:10,000, LI-COR Biosciences, Lincoln, NE, USA) or HRP goat anti-mouse IgM antibody (1:10,000, Thermo scientific, Waltham, MA, USA). After incubation for 1 h at room temperature in a light-proof container, followed by thorough washing, the membranes were visualized by a LI-COR Odyssey^®^ Infrared Imaging System (LI-COR Biosciences) or ImageQuant LAS 4000 mini system (GE Healthcare, Chicago, IL, USA).

### 2.4. Isolation of OMVs

OMVs were isolated from the supernatant of bacterial culture using a method described by Wai et al., with minor modifications [39]. Briefly, bacterial suspensions obtained from different strains of *H. pylori* harvested at the stationary phase (about 60 h of bacterial growth, the value of optical density at 600 nm (OD_600_) ≌ 2.0) in a total volume of 12.5 mL were centrifuged at 4000× *g* for 10 min at 4 °C. The obtained supernatants were filtered with 0.45 μm membranes and then ultracentrifuged at 20,000× *g* for 2 h at 4 °C to collect the OMVs (Micro Ultracentrifuge CS150NX, Hitachi Instruments Inc., Tokyo, Japan). The pellets containing OMVs were washed twice with PBS and resuspended in 20 mM Tris-HCl (pH 8.0) for further analysis.

### 2.5. LPS Isolation and LPS Profile Analysis

LPS from the tested *H. pylori* strains was isolated by the hot phenol–water method, with minor modifications [40]. In brief, different strains of *H. pylori* cultured in 1.5 mL of Brucella broth media at the log phase (OD_600_ ≌ 1.0) were harvested by centrifugation (4000× *g* for 10 min at 4 °C), and the whole-cell lysates containing LPS were extracted from the pellets by adding 600~800 μL of lysis buffer. One hundred and fifty microliters of the whole-cell lysate was added with 10 μL of proteinase K (20 mg/mL), and the resulting suspension was incubated at 60 °C for 120 min. Then, 150 μL of the prewarmed 90% hot phenol solution (70 °C) was added to the suspension and incubated in a water bath at 70 °C for 15 min with constant shaking or vortexing. Thereafter, the mixture was allowed to cool down on ice for 10 min and then subjected to centrifugation (4000× *g* for 10 min at 4 °C). After centrifugation, LPS was recovered from the aqueous phase. For silver staining, the purified LPS was separated on 15% trincine gel. The obtained gel was first fixed overnight in 150 mL of the fixing solution (25% isopropanol and 7% acetic acid), followed by incubation with the oxidation solution containing 7.5% acetic acid and 0.7% periodic acid for 10 min. The gel was then washed three times for 30 min each with distilled water, and stained with 150 mL of a freshly prepared staining solution (28 mL of 0.1 N NaOH, 2 mL of ammonium hydroxide and 5 mL of 20% silver nitrate) for 25 min. After staining, the gels were washed three times for 10 min each with distilled water and developed by reduction in 200 mL of the developing solution (0.05% citric acid and 0.074% formaldehyde) until the signal of LPS became visible.

### 2.6. Growth Curve Analysis of H. pylori

Various *H. pylori* strains were first grown on blood agar plates for 48 h, and then switched to Brucella broth media with 10% FBS (Biological Industries) and 1% IsoVitale X (Creative) under microaerophilic conditions at 37 °C for 16 h. The bacterial cultures were later diluted to an OD_600_ value of 0.01 and incubated with shaking at 140 r.p.m. under microaerophilic conditions for up to 3 days. The value of OD_600_ was measured by spectrophotometer at various time points, and the experiment was repeated in triplicate to obtain the averaged results and standard deviation.

### 2.7. SDS and Novobiocin Sensitivity Assay

Bacteria were harvested and diluted to an OD_600_ value of 0.6 in Brucella broth media and 100 μL of cell suspension was added to 1900 μL of Brucella broth medium supplemented with an appropriate amount of the detergent SDS or the antibiotic novobiocin, with concentrations of up to 0.02% for SDS and 100 μg /mL for novobiocin. The assays were executed in triplicate with independent cultures in 24-well plates with shaking at 140 r.p.m. under microaerophilic conditions at 37 °C, and the OD_600_ values were measured after 72 h of bacterial growth. The survival rate was defined as follows: survival rate (%) = (OD_600_ of treatment/OD_600_ of control) × 100.

### 2.8. Bacterial Infection Assay

AGS cells (ATCC 1739, human gastric adenocarcinoma epithelial cell line) were seeded at 2 × 10^5^ cells/well in 6 cm dishes containing Ham’s F-12 media (Sigma-Aldrich) supplemented with 10% FBS under standard cell culture conditions. When the AGS cells reached approximately 80% confluency, suspensions from different strains of *H. pylori* harvested at the log phase (OD 600 ≌ 1.0) were added to the AGS cells at a multiplicity of infection (MOI) of 100. After 6 h of co-culturing the morphological changes of the cells were recorded by using an Olympus IX71 research inverted system microscope (Olympus, Tokyo, Japan) with 20× magnification.

### 2.9. Adhesion Assay

Following the bacterial infection described above, the dish with the AGS cell–bacterium co-culture was washed three times with warm Ham’s F-12 media without FBS and mildly shaken to remove non-adherent bacteria. The AGS cells were subsequently lysed with 1 mL of PBS buffer containing 0.1% saponin to release the cell-associated bacteria. The obtained lysate was serially diluted and spread on sheep blood agar plates. The adhered bacteria were measured by the viable plate counting method after 72 to 96 h of incubation.

### 2.10. Internalization Assay

Following the bacterial infection described above, the dish with the AGS cell–bacterium co-culture was washed with a PBS buffer and the medium was then replaced by Ham’s F12 medium with 100 μg/mL gentamicin for 1 h of incubation to kill the rest of the surface-associated, non-internalized bacteria. After incubation, the dish was washed with a PBS buffer and the medium was then replaced by Ham’s F12 medium with 10 μg/mL gentamicin. The infected cells were further incubated for 24 h under standard culturing conditions. After incubation, cells were lysed with a 0.5% saponin-containing PBS buffer for 5 min. The lysate was then serially diluted and spread on sheep blood agar plates. The internalized bacteria were measured by the viable plate counting method after 72 to 96 h of incubation.

### 2.11. The Surface Hydrophobicity and Autoaggregation Assays

Bacteria were grown in Brucella broth media with 10% FBS and 1% IsoVitale X under microaerophilic conditions at 37 °C for 48 h. For the surface hydrophobicity assay, bacteria were harvested and diluted to reach an OD_600_ value of 1.0 in a tube containing 5 mL of phosphate urea magnesium sulfate buffer (PUM: 22.2 g/L K_2_HPO_4_-3H_2_O, 7.26 g/L KH_2_PO_4_, 1.8 g/L urea, 0.02 g/L MgSO_4_-7H_2_O; pH 7.1) supplemented with 400 μL of n-hexadecane. The mixture was vortexed for 2 min to homogenize the bacterial suspension, and the tube containing bacteria was then left on the bench and stood up for 24 h. At the start of the experiment, the OD_600_ of the bacterial suspension was measured as A_0_. During the test, 500 μL of solution from the top of the bacterial suspension was collected at 6, 8 and 24 h, and followed by measuring the OD_600_ value as A_t_. The hydrophobicity index was defined as follows: the hydrophobicity index = (A_0_ − A_t_)/A_0._ For the autoaggregation assay, bacteria were harvested and diluted to reach an OD_600_ value of 1.0 in a tube containing 6 mL of PBS, and the tube was then left on the bench and stood up for 24 h. The collection of bacterial suspension and the OD_600_ recording were executed as described for the surface hydrophobicity assay. The ratio of autoaggregation was defined as follows: the autoaggregation rate (%) = (A_0_ − A_t_)/A_0_ × 100_._ Each experiment was repeated in triplicate to obtain the average and standard deviation.

### 2.12. Statistics Analysis

Statistical analyses were performed using an unpaired, two-tailed Student’s *t*-test. Significant differences were identified at * *p* < 0.05, ** *p* < 0.01 and *** *p* < 0.001.

## 3. Results

### 3.1. Sequence Analysis of the HP0044 and HP1275 Proteins in H. pylori 26695

**Figure 2:**
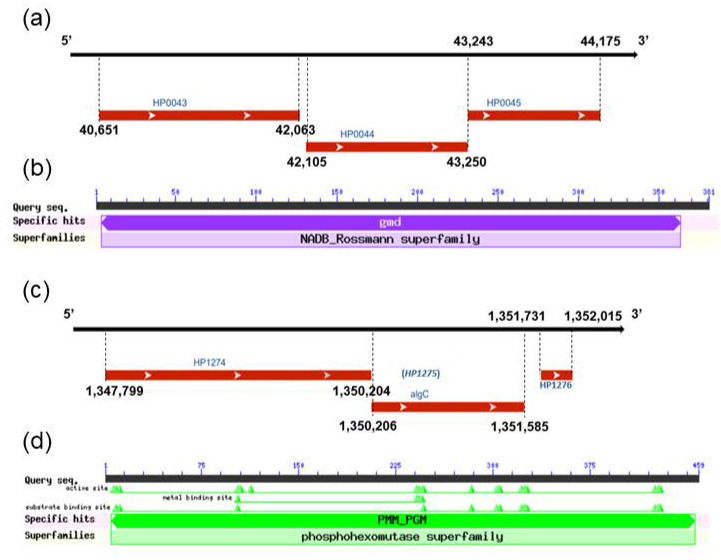
The bioinformatic analysis of the *HP0044* and *HP1275* genes and the encoded proteins in *H. pylori* wild-type strain 26695. (**a**) The gene locus of *HP0044*. (**b**) The prediction of conserved domains in the HP0044 protein. (**c**) The gene locus of *HP1275*. (**d**) The prediction of conserved domain in the HP1275 protein. The data were obtained from the NCBI (https://www.ncbi.nlm.nih.gov/, accessed on 11 December 2021).

The *HP0044* coding sequence is located in the plus strand from 42,105 bps to 43,250 bps of the *H. pylori* genomic DNA, and is flanked by the *HP0043* and *HP0045* genes (Figure 2a). Similarly, the *HP1275* coding sequence is located in the plus strand from 1,350,206 bps to 1,351,585 bps of the *H. pylori* genomic DNA, and is flanked by the *HP1274* and *HP1276* genes (Figure 2c). The *HP0044* gene is 1146 bps in length, and can be translated into a protein with 381 amino acid residues. As for *HP1275* gene, it contains 1380 bps and can be translated into a protein with 459 amino acid residues. The conserved domain of the HP0044 protein is found in the members of the NADB_Rossmann superfamily, which have the Rossmann-fold NAD(P)H/NAD(P)^+^ binding (NADB) domain, and the conserved domain of the HP1275 protein belongs to the phosphohexomutase superfamily, which catalyzes a reversible intramolecular phosphoryl transfer of the sugar substrates (Figure 2b,d).

The amino acid sequences of the HP0044 and HP1275 proteins and the GMD, PMM and PGM from different species of Gram-negative bacteria were aligned by using the LALIGN program, and the results are summarized in Table 3. Based on the results obtained from the sequence alignment, the HP0044 protein displays a high degree of sequence homology with GMD in other Gram-negative bacteria (61.7% identity for *Yersinia enterocolitica* 8081, 60.1% identity for *Escherichia coli* K12, 59.6% identity for *Salmonella typhimurium* CT18 and 61.0% identity for *Vibrio cholerae* O1 biovar El Tor N16961). Similarly, the HP1275 protein also showed a certain degree of homology with PMM and PGM in other Gram-negative bacteria (28.2% identity of PMM/PGM for *Xanthomonas campestris pv. Vesicatoria*, 38.9% identity of PMM/PGM for *Pseudomonas aeruginosa* PAO1, 27.4% identity of PMM for *Salmonella typhimurium* SL1344, 26.4% identity of PMM for *Vibrio cholerae *2017V-1105**, 21.6% identity of PGM for *V. cholerae* and 40.4% identity of PGM for *Neisseria gonorrhoeae*). This result indicated that the HP0044 protein could be a putative GMD that catalyzes the conversion of GDP-mannose into GDP-4-dehydro-6-deoxy-D-mannose, and the HP1275 protein could be a putative PMM/PGM that catalyzes the interconversion between α-D-mannose-6-phosphate and α-D-mannose-1-phosphate as well as α-D-glucose-6-phosphate and α-D-glucose-1-phosphate. The two enzymes could be involved in the biosynthesis of fucose and LPS in *H. pylori* (Figure 1).

### 3.2. The HP0044 and HP1275 Proteins Are Critical for LPS Expression

To understand the role of the HP0044 and HP1275 proteins in *H. pylori*, the *HP0044* and *HP1275* knockout mutants and their corresponding knockout complementary mutants were constructed. The *HP0044* and *HP1275* genes in the *H. pylori* genomic DNA were disrupted by the insertion of a chloramphenicol resistance cassette (${\mathrm{Cm}}^{r}$) to generate the knockout mutants, and the resulting mutants were complemented by the insertion of a DNA fragment containing the promoter region of the *HP1563* gene and the ORFs of the *HP0044* or *HP1275* gene into the *rdxA* gene region to generate the knockout complementary mutants. The successful generation of these mutants was verified by PCR (data not shown).

**Figure 3:**
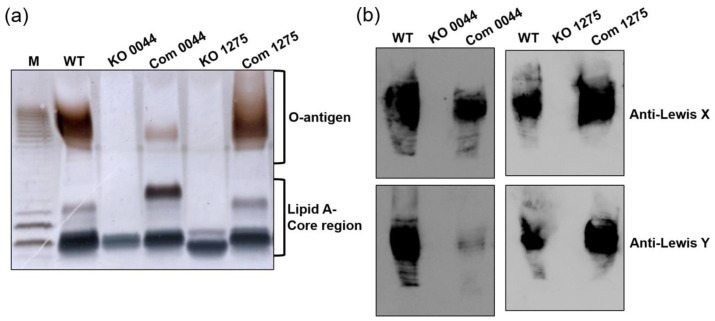
Effects of the *HP0044-* and *HP1275*-disrupted mutations on LPS profile and Lewis antigen presentation. The LPS profiles from wild-type strain 26695, the *HP0044* and *HP1275* knockout mutants and their corresponding knockout complementary mutants were analyzed by (**a**) 15% tricine-PAGE with silver staining or (**b**) immunoblotting using anti-Lewis X and anti-Lewis Y antibodies. The relative positions of the O-antigen and lipid A–core region were indicated by braces. M, *E. coli* O111:B4 LPS marker; WT, wild-type strain; KO, knockout mutant; and Com, knockout complementary mutant.

The recently redefined LPS structure in *H. pylori* is relatively linear, and the fucose residues appear not only in the O-antigen but also in the “Trio” region, which connects the core oligosaccharide to the LPS O-antigen. This suggests that a lack of fucose residues may lead to a truncated LPS structure shorter than that being considered previously. To explore the involvement of fucose in the construction of LPS, the effect of the *HP0044*- and *HP1275*-disrupted mutations on LPS expression was evaluated by silver staining and immunoblotting analysis to examine the LPS profile and Lewis antigen moieties, respectively. As shown in Figure 3a, the complete loss of the O-antigen and an aberrant core region were observed in both *HP0044* and *HP1275* knockout mutants using tricine gel with silver staining. Furthermore, while testing with immunoblotting analysis to investigate the presence of Lewis antigens, the signals of Lewis X and Lewis Y antigens completely disappeared in both *HP0044* and *HP1275* knockout mutants (Figure 3b). The LPS truncation was fully restored in the *HP1275* knockout complementary mutant while the *HP0044* knockout complementary mutant showed slight abnormalities in silver staining and immunoblotting analysis, suggesting that the complementation of the *HP0044* gene is not complete, possibly due to the lower level of expression of the complemented HP0044 protein. Interestingly, a slight difference between the LPS core region of the *HP1275* knockout mutant and that of the *HP0044* knockout mutant was also observed in Figure 3a. The reason for the difference between the LPS profiles of these two knockout mutants could result from another activity aquired by the HP1275 protein, which may potentially preform the enzymatic function of PGM, associating with the biosynthesis of glucose, which is also present in the side chain of the LPS core region in *H. pylori* (Figure 1). In summary, our results support the newly proposed LPS structure, that the fucose residue is involved in the “Trio” region of the LPS O-antigen in *H. pylori* wild-type strain 26695.

### 3.3. HP00044/HP1275 Gene Disruptions Affect Bacterial Growth, Sensitivity to the Detergent SDS and Resistance to the Antibiotic Novobiocin

**Figure 4:**
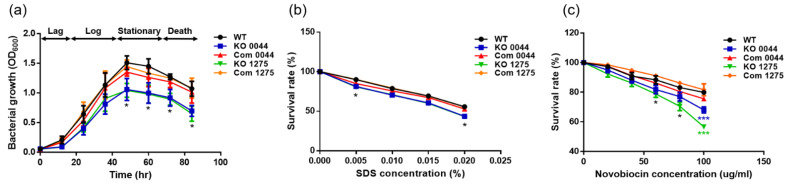
Effects of the *HP0044-* and *HP1275*-disrupted mutations on *H. pylori* growth, sensitivity to the detergent SDS and resistance to the antibiotic novobiocin. (**a**) The growth curves of wild-type strain 26695, the *HP0044* and *HP1275* knockout mutants and their corresponding knockout complementary mutants. The data were recorded by a spectrophotometer at ${\mathrm{OD}}_{600}$ at different time points. The data are presented as the average ${\mathrm{OD}}_{600}$ values derived from triplicate trials with statistical analysis (unpaired, two-tailed Student’s *t*-test; * *p* < 0.05). (**b**) SDS sensitivity and (**c**) novobiocin resistance of the tested *H. pylori* strains. The effects of the *HP0044* and *HP1275* mutations on SDS sensitivity and novobiocin resistance of *H. pylori* were evaluated by the survival rate, which was measured by the ratio of the ${\mathrm{OD}}_{600}$ of bacteria incubated with/without various concentrations of SDS and novobiocin. The data presented were derived from triplicate trials with statistical analysis (unpaired, two-tailed Student’s *t*-test; * *p* < 0.05 and *** *p* < 0.001). WT, wild-type strain; KO, knockout mutant; and Com, knockout complementary mutant.

To evaluate the impact of the *HP0044-* and *HP1275*-disrupted mutations on the growth of *H. pylori*, wild-type strain 26695, the *HP0044* and *HP1275* knockout mutants and the corresponding knockout complementary mutants were subjected to a growth assay. The growth curves were generated by recording the OD_600_ of bacteria, starting at an OD_600_ of 0.05 in Brucella broth supplemented with 10% FBS under microaerophilic conditions at 37 °C for 84 h. The data were recorded by a spectrophotometer for every 12 h within this period. The results showed that both *HP0044* and *HP1275* knockout mutants had a slight delay in the lag phase of bacterial growth and grew slower than wild-type strain 26695 and the corresponding knockout complementary mutants (Figure 4a). However, all of the tested strains reached their maximal culture densities after 48 h of growth and showed similar durations in each phase of bacterial growth. This observation suggested that the loss of either the HP0044 or HP1275 proteins can hamper *H. pylori* growth.

Detergent sensitivity and antibiotic resistance are important determinants for the survival of bacteria in harsh environments. LPS on the OM is pivotal for Gram-negative bacteria to protect themselves from the damage of xenobiotics. Therefore, defective LPS may make bacteria lose their ability to withstand the stress exerted by toxic molecules, and thus die. Due to the importance of LPS in bacterial protection, SDS sensitiviy and novobiocin resistance assays were conducted to evaluate the impacts of the *HP0044-* and *HP1275*-disrupted mutations on the susceptibility of *H. pylori* to detergents and hydrophobic antibiotics. As shown in Figure 4b,c, the survival rates of the *HP0044* and *HP1275* knockout mutants to SDS and novobiocin were both decreased more significantly than those of wild-type strain 26695 at various tested concentrations, suggesting that the tolerance of the knockout mutants to these two xenobiotics was compromised. As for the corresponding LPS-related knockout complementary mutants, the survival rates were restored to levels similar to those of wild-type strain 26695. The results indicated that the HP0044 and HP1275 proteins are required for the biosynthesis of LPS and thus the maintainence of the membrane integrity which is critical for bacterial protection against toxic molecules in harsh environmental conditions.

### 3.4. The HP0044- and HP1275-Disrupted Mutations Significantly Affect H. pylori Membrane Properties

**Figure 5:**
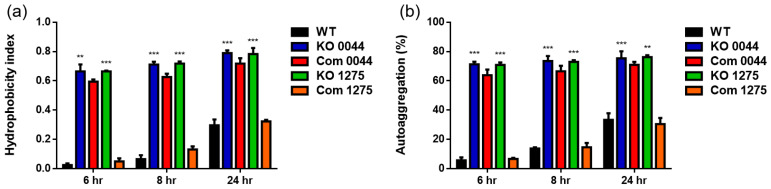
Effects of the *HP0044-* and *HP1275*-disrupted mutations on the membrane properties of *H. pylori*. Various *H. pylori* strains were incubated with (**a**) an organic solvent, n-hexadecane, for the surface hydrophobicity analysis, and (**b**) phosphate-buffered saline (PBS) for the autoaggregation assay. The hydrophobicity index and autoaggregation percentage, representing the cell surface hydrophobicity and bacterial autoaggregation, respectively, were calculated from the OD_600_ values recorded at different time points of the corresponding experiments, as described in the Materials and Methods section. The OD_600_ value obtained from the zero time point served as a control for background subtraction. Data presented are derived from triplicate trials with statistical analysis (unpaired, two-tailed Student’s *t*-test; ** *p* < 0.01 and *** *p* < 0.001). WT, wild-type strain; KO, knockout mutant; and Com, knockout complementary mutant.

The effects of the *HP0044*- and *HP1275*-disrupted mutations on the bacterial membrane properties were examined by treating the tested *H. pylori* strains with an organic solvent and PBS for the surface hydrophobicity analysis and autoaggregation assay, respectively. The results showed that the surface hydrophobicity of both the *HP0044* and *HP1275* knockout mutants significantly increased at all of the time points tested (6 h, 8 h and 24 h) compared to that of wild-type strain 26695 (Figure 5a). A similar trend was also observed when the bacterial autoaggregation was measured (Figure 5b). The *HP1275* knockout complementary mutant displayed a similar degree of surface hydrophobicity and bacterial autoaggregation compared to that of wild-type strain 26695, while the *HP0044* knockout complementary mutant only showed a partial level of restoration. This observation agrees very well with the results obtained in the LPS profile and Lewis antigen analyses, indicating that the incomplete complementation of the *HP0044* gene in the *HP0044* knockout complementary mutant currently generated cannot fully recover all of the functions or structures that the fucose residues are involved in.

### 3.5. The HP0044- and HP1275-Disrupted Mutations Have Profound Influences on Bacterial Virulence

**Figure 6:**
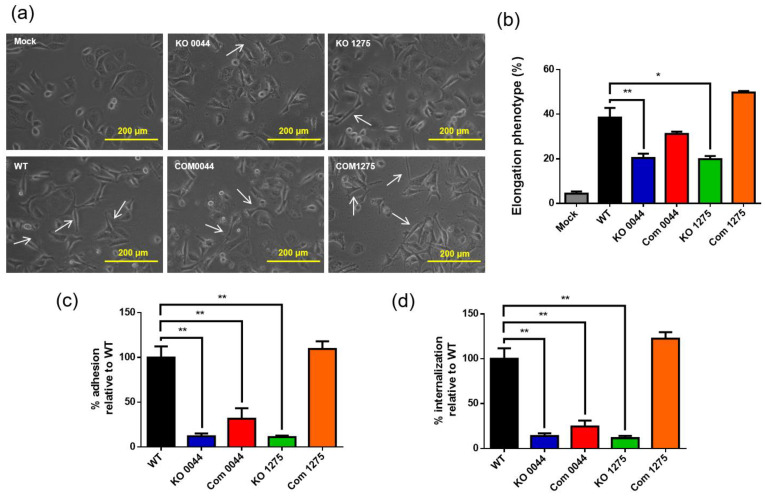
Effects of the *HP0044-* and *HP1275*-disrupted mutations on bacterial virulence. (**a**) Morphological changes of the AGS cells infected by different *H. pylori* strains. The morphological changes of the AGS cells after *H. pylori* infection were documented by phase-contrast microscopy with 20× magnification. The AGS cells exhibited long spindles and an elongated phenotype, indicated by arrows. (**b**) Quantitation of the AGS cells with the elongated phenotype after *H. pylori* infection. The percentage of elongated cells was quantified from 10 different 0.25 mm^2^ fields. The data presented were derived from triplicate trials with statistical analysis (unpaired, two-tailed Student’s *t*-test; * *p* < 0.05 and ** *p* < 0.01). (**c**) Adhesion of the AGS cells by different strains of *H. pylori* infection. Various *H. pylori* strains were co-incubated with the AGS cells at an MOI (multiplicity of infection) of 100. The adhesion of different mutants measured by the viable plate counting method was compared to that of wild-type strain 26695 to obtain the relative percentage of bacterial adhesion. The data presented were derived from triplicate trials with statistical analysis (unpaired, two-tailed Student’s *t*-test; * *p* < 0.05 and ** *p* < 0.01). (**d**) Internalization of the AGS cells by different strains of *H. pylori* infection. Various *H. pylori* strains were co-incubated with the AGS cells at an MOI of 100. The internalization of various knockout mutants measured by the viable plate counting method was statistically compared to that of wild-type strain 26695 to obtain the relative percentage of bacterial internalization. The data presented were derived from triplicate trials with statistical analysis (unpaired, two-tailed Student’s *t*-test; ** *p* < 0.01). WT, wild-type strain; KO, knockout mutant; and Com, knockout complementary mutant.

Studies have shown that the morphology of human gastric adenocarcinoma (AGS) cells would alter after *H. pylori* infection, and that the infected cells display a typical elongated and spreading appearance called the “hummingbird phenotype”. To elucidate on whether the *HP0044-* and *HP1275*-disrupted mutations would affect the outcomes of bacterial infection, a cell infection assay was conducted. When the AGS cells were infected by wild-type strain 26695, the classical hummingbird phenotype indeed appeared (Figure 6a). Although the hummingbird phenotype of the AGS cells could also be observed when the AGS cells were infected with the *HP0044* and *HP1275* knockout mutants, the percentage of the AGS cells showing the hummingbird phenotype was significantly lower than that of the cells infected by wild-type strain 26695 (20% compared to 38.5%, Figure 6b). The appearance of the hummingbird phenotype of the infected AGS cells was restored when the cells were infected by the *HP1275* knockout complementary mutant, while the restoration was less significant by the infection of the *HP0044* knockout complementary mutant. These results suggested that the loss of the HP0044 and HP1275 proteins could lead to a reduction in the infection ability of *H. pylori*, thus indicating that the intactness of *H. pylori* LPS is highly correlated with the virulence of *H. pylori*.

Previous studies have reported that LPS is associated with the adhesion ability of many Gram-negative bacteria [33,34,35,41]. However, the involvement of LPS in *H. pylori* colonization is under debate. Therefore, bacterial adhesion and internalization assays were conducted to investigate the role of LPS in *H. pylori* colonization. As shown in Figure 6c, the disruption of the *HP0044* and *HP1275* genes significantly suppressed the adhesion ability of *H. pylori*. The *HP0044* knockout complementary mutant showed partial restoration of the bacterial adhesion ability and the *HP1275* knockout complementary mutant displayed full restoration of the bacterial adhesion ability compared to wild-type strain 26695, which was in line with most of results of the functional and characteristic analyses conducted in this study. Consistent with the result of the adhesion assay, the relative percentage of the internalized *HP0044* and *HP1275* knockout mutants in the AGS cells was also reduced significantly compared to that of wild-type strain 26695 (Figure 6d). In terms of the complementary strains, the *HP0044* knockout complementary mutant still possessed less internalization ability, but the *HP1275* knockout complementary mutant had similar internalization ability to that of the wild-type strain. The results suggested that a defect in the biosynthesis of fucose and thus the LPS structure would have a profound influence on *H. pylori* colonization.

### 3.6. HP0044 and HP1275 Gene Disruptions Affect Protein Sorting into H. pylori OMVs

**Figure 7:**
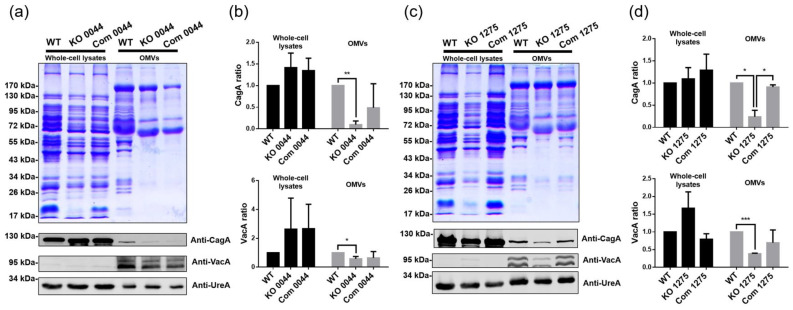
Effects of the *HP0044-* and *HP1275-*disrupted mutations on protein sorting into OMVs. (**a**,**c**), comparison of protein profiles and CagA, VacA and UreA protein levels between the whole-cell lysates and OMVs in different strains of *H. pylori*. (**b**,**d**), statistical analysis of the CagA and VacA content. CagA, VacA and UreA were quantified using ImageJ software and normalized to UreA. The data presented were derived from triplicate trials with statistical analysis (unpaired, two-tailed Student’s *t*-test; * *p* < 0.05, ** *p* < 0.01 and *** *p* < 0.001). Proteins from the whole-cell lysates and OMVs were analyzed by 15% SDS-PAGE with Coomassie blue staining and immunoblotted using anti-CagA, anti-VacA and anti-UreA antibodies. WT, wild-type strain; KO, knockout mutant; and Com, knockout complementary mutant.

Previous studies have shown that the alteration of protein sorting into OMVs is associated with the biosynthesis of LPS in some bacteria, including *H. pylori* [35,42]. To test whether the biosynthesis of fucose is also involved in the control of the protein-sorting mechanism in *H. plori*, proteins extracted from both bacteria and OMVs were collected from the tested *H. pylori* strains and subjected to subsequent Commassie Blue staining and immunoblotting analyses. Figure 7 shows a dramatic difference between the protein contents from the whole-cell lysates and OMVs, indicating that only a portion of specific proteins can be sorted into or even enriched in OMVs. The protein profiles of the whole-cell lysates were similar among wild-type strain 26695, the *HP0044* and *HP1275* knockout mutants and the corresponding knockout complementary mutants, while a significant difference could be seen in the protein profiles of OMVs between wild-type strain 26695 and the knockout mutants. Many proteins enriched in the OMVs of wild-type strain 26695 were reduced or even completely disappeared in those obtained from the knockout mutants. Specifically, virulence factors UreA, CagA and VacA were chosen for the immunoblotting analysis. The results showed that the amount of CagA and VacA was significantly reduced in the OMVs obtained from the *HP0044* and *HP1275* knockout mutants, while the amount of UreA remained similar among the tested strains. The protein profiles of OMVs and the amount of CagA and VacA were not significantly recovered in the *HP0044* knockout complementary mutant compared to those of the *HP0044* knockout mutant, which is in accordance with the previous observation that the complementary ability of the *HP0044* knockout complementary mutant is incomplete. These results suggested that certain proteins in *H. plori* can be specifically sorted into the OMVs, and that the HP0044 and HP1275 proteins involved in the biosynthesis of fucose of LPS play an important role in affecting the protein-sorting mechanism of *H. pylori*.

## 4. Discussion

LPS plays an important role in Gram-negative bacteria, maintaining the membrane integrity, forming an impermeable barrier, influencing the host immune responses and promoting bacterial adhesion. It also serves as a key virulence factor for the long-term colonization of *H. pylori* in the harsh gastric environment [16,43]. The terminus of the O-antigen in *H. pylori* is usually decorated by fucose and forms the structure of Lewis X and Lewis Y antigens. The Lewis antigens on the *H. pylori* O-antigen serve as a form of molecular mimicry because similar or even identical glycan structures, including Lewis X/Y and Lewis a/b antigens, can be found on the epithelial cells in the human stomach. This similarity provides an advantageous strategy for *H. pylori* to evade host immune surveillance and enables this bacterium to persist in the stomach [19].

In the past, the structure of LPS in *H. pylori* was considered to be a branched form, and the O-antigen was attached to the inner core of LPS as a side chain (Figure 1a). According to this branched LPS structure, fucose residues only exist in the terminus of the LPS O-antigen. Recently, the LPS structure in *H. pylori* wild-type strains G27 and 26695 was re-examined [13]. The results showed that the core oligosaccharide is shorter than the one previously recognized, and that the O-antigen is connected to the core oligosaccharide in a linear form (Figure 1a). The most significant change in this newly proposed LPS structure is the presence of a fucose residue in the “Trio” region that directly connects to the core region of LPS. The existence of fucose in the LPS backbone potentially increases the importance of fucose in the structure and function of *H. pylori* LPS. Previous reports have demonstrated that GMD and PMM are necessary for the synthesis of a complete LPS structure in *Pseudomonas* species and *Shigella flexneri* [32,44,45]. In the current study, two genes, *HP0044* and *HP1275*, which are homologous to GMD and PMM/PGM, respectively, and predicted to be associated with the biosynthesis of fucose in wild-type strain 26695, were knocked out to generate the corresponding mutants. The LPS profiles and physiological traits of these knockout mutants were also characterized, showing that a defect in the biosynthesis of fucose in the *HP0044* and *HP1275* knockout mutants not only causes the disappearance of the Lewis antigens on the LPS terminus but also leads to a significant loss in the entire O-antigen polysaccharide (Figure 3). In addition, the amount and position of the core oligosaccharide in the knockout mutants also show significant alterations, thus suggesting that a deficiency in the biosynthesis of fucose may impose more complex influences on the biosynthesis of LPS in *H. pylori* originally thought. According to the data presented, the fucose is indeed present in the backbone of the LPS O-antigen of *H. pylori* wild-type strain 26695, which supports the *H. pylori* LPS structure newly proposed by Li et al. [13].

Our group has previously demonstrated that *HP0857*, *HP0859* and *HP0860* are all essential for the biosynthesis of heptose, which is involved in the LPS core region of *H. pylori* [33,34,35]. Disruptions of the *HP0857*, *HP0859* and *HP0860* genes not only cause the loss of a major part of the LPS structure in *H. pylori* but also result in an increase in its susceptibility to the hydrophobic antibiotic novobiocin and the detergent SDS. Furthermore, these knockout mutants all show a dramatic decline in the adhesion ability to AGS cells and thus cause less “hummingbird” phenotypes of AGS cells after bacterial infection. In this study, we further demonstrated that LPS truncation caused by fucose deficiency can cause a significant decrease in bacterial membrane permeability, adherence and internalization ability and virulence, which echoes our previous findings on the biosynthesis of heptose in *H. pylori* about the importance of LPS in the fitness and virulence of *H. pylori*. It is worth noting that similar results are also found in other bacteria. Chiku et al. showed that the deficiency of GMD affects the structure of LPS, bacterial motility and cell surface hydrophobicity in *Pseudomonas syringae* Pathovar *glycinea* Race 4 [45]. Tang et al. reported that the *algC* knockout mutation can decrease the virulence of *P. aeruginosa* [46], and that the ability of the resultant mutant to cause bacteriemia and death in a neonatal mouse model of infection is significantly reduced compared to that of wild-type strain PAO1. Taken together, our findings agree with others in certain bacteria and suggest that the deficiency of GMD and PMM/PGM and thus the loss of an intact LPS structure could lead to profound defects in the fitness and virulence of *H. pylori*.

Bacterial biofilms are composed of communities of multiple bacterial species with various self-produced extracellular polymeric substances (EPS), including proteins, nucleic acids, carbohydrates and OMVs, which together are attached to biotic or abiotic surfaces. Research has demonstrated that *H. pylori* is capable of forming biofilms in response to different environmental stresses and for adaptating to the gastric niche as well as host colonization [47]. The formation of *H. pylori* biofilms can alleviate susceptibility to antimicrobial agents, which may lead to the failure of *H. pylori* eradication therapy and increase the medical burden [48]. LPS was found to be one of the major macromolecules in the *H. pylori* biofilm matrix [49]. Some *H. pylori* genes related to LPS biosynthesis and export have been shown to be associated with the transition from the planktonic to the biofilm phase of bacterial growth [50,51,52]. Both bacterial surface hydrophobicity and autoaggregation are key factors for the development of biofilm [53]. Interestingly, a recent study showed that the formation of biofilm in *H. pylori* is regulated by a two-component acid response system (ArsRS) [54]. Mutations in the ArsRS system not only increased the level of bacterial autoaggregation but also enhanced the formation of biofilm, thus revealing the correlation between these two phenotypes in *H. pylori* and the involvement of environmental pH in the modulation of the development of biofilm. In the present study, the surface hydrophobicity of *H. pylori* is significantly increased and the bacterium is prone to autoaggregation when the biosynthesis of fucose is out of order. Accordingly, it is appealing to presume that the mutations of HP0044 and HP1275 may have a positive effect on promoting the formation of biofilm. The fact that the HP0044 and HP1275 mutations can reduce the bacterial virulence associated with toxicity, but that they may also enhance the development of biofilm by *H. pylori*, has made a further study aiming at revealing the importance of the biosynthesis of fucose in the formation of biofilm in *H. pylori* and its clinical impact an interesting topic to explore.

The production of OMVs budding from the outer membrane of most Gram-negative bacteria can also be found in *H. pylori* [55,56]. Accumulating evidence has suggested that the formation of OMVs is a general strategy for Gram-negative bacteria to export certain kinds of proteins to the environment or the host cells [57]. In addition, OMVs play mutiple roles in promoting bacterial adhesion [58], invasion [59] and the formation of biofilm [60], enhancing antibiotic resistance for the whole community [61], causing damage to the host cells through toxin transport [62] and modulating the host immune responses by regulating the proteins in the host immune system [63]. Research has shown that the composition of proteins in the OMVs is different from that in the cytoplasm, periplasm and OM; however, the detailed mechanism for the process of protein enrichment or exclusion is still not clear [64,65]. Previously, Haurat et al. demonstrated that the protein-sorting process is associated with the biosynthesis of LPS in *Porphyromonas gingivalis* [42]. The loss of the LPS O-antigen in *P*. *gingivalis* resulted in an aberrent protein presentation in OMVs. Alterations in the protein composition in OMVs due to a loss of the O-antigen has been reported in *Klebsiella pneumoniae* as well [66]. Similar results were also found in our previous study, which showed that a gene-disrupted mutation affecting the biosynthesis of heptose not only caused a severe truncation of LPS but also altered the protein profile of OMVs [35]. In addition, an important virulence factor, CagA, was nearly absent from the OMVs of the derived heptose-deficient mutant [35]. In the current study, we further confirmed the relationship between the biosynthesis of LPS and the mechanism of protein sorting into OMVs. The protein profile of OMVs from the *HP0044* and *HP1275* knockout mutants, which displayed an O-antigen-deficient phenotype, showed significant alterations compared to that from wild-type strain 26695. Notably, not only CagA but also VacA, another key virulence factor of *H. pylori*, was substantially reduced in these knockout mutants. The data presented in this study are in agreement with our previous findings and imply that the integrity of LPS is associated with the selective mechanism of sorting proteins into OMVs in *H. pylori*.

In this study, most of the data presented indicated that the *HP0044* knockout complementary mutant does not have its GMD function fully restored. Only a faintish signal of the LPS O-antigen can be observed in the *HP0044* knockout complementary mutant from the silver staining of the LPS profile and the Lewis Y antigen staining (Figure 3). Similar phenomena also appear in the data obtained from the membrane property assays (Figure 5), the bacterial infection assays (Figure 6) and the assays for protein sorting into OMVs (Figure 7). These abnormal results may be related to the poor efficiency of *HP0044* gene complementation. This inefficiency could have resulted from (1) polar effects that occurred during the homologous recombination of the *HP0044* gene to the *RdxA* locus in the genomic DNA of the *HP0044* knockout mutant, or (2) an inappropriate choice of the promoter sequence for the expression of the *HP0044* gene. In contrast, the *HP1275* knockout complementary mutant was shown to be comparable to wild-type strain 26695 in all of the tests. Although the complementary efficiency of the allotopic *HP0044* gene was lower than expected, both the *HP0044* and *HP1275* knockout complementary mutants can still restore the functions of the corresponding knockout mutations to a certain degree, thus confirming that the *HP0044* and *HP1275* mutations indeed contribute to the alterations observed in this study.

In summary, we demonstrated that disruptions of the *HP0044* and *HP1275* genes could lead to a deficiency in fucose production, which would further affect the biosynthesis of LPS. Our data also supported the newly proposed LPS structure that, in addition to decorating the terminus of the LPS O-antigen to form Lewis antigen moieties, posited that fucose is also involved in a “Trio” region of the O-antigen, connected to the core oligosaccharide in *H. plyori*. A defect in the production of fucose caused a loss of a major part of LPS in *H. pylori*, and thus resulted in a slight retardation in bacterial growth and higher susceptibility to the detergent SDS and the hydrophobic antibiotic novobiocin. In addition, a truncated LPS without the O-antigen increased the surface hydrophobicity of *H. pylori* and promoted bacterial autoaggregation. Furthermore, fucose deficiency also significantly affected bacterial virulence and protein sorting into OMVs of *H. pylori*. The findings aquired demonstrated that GMD and PMM/PGM in *H. pylori* are potentially important for bacterial fitness and pathogenesis. Therefore, these enzymes can be candidates for the development of drugs against *H. pylori* infection, and further researche on this aspect is worth conducting.

## Figures and Tables

**Table 1:** Bacterial strains and plasmids used in this study.

Strain/Plasmid		Characteristics and Marker ^1^	Source
Strain	*E. coli*		
	Top10	The host for construction of pGEM-T-HP0044 and pGEM-T-HP1275 clones	Invitrogen, Carlsbad, CA, USA
	DH5α	The host for construction of pGEM-T-HP0044^d^::Cm^r^ and pGEM-T-HP1275^d^::Cm^r^ clones	Invitrogen, Carlsbad, CA, USA
	*H. pylori*		
	26695	*H. pylori* whole-genome sequencing strain, isolated from the stomach of a patient with gastritis	ATCC ^2^ 700392
	KO 0044	*H. pylori* 26695 strain with a chloramphenicol resistance cassette in *HP0044*; Cm^r^	This study
	Com 0044	KO 0044 with *HP0044* insertion in *HP0954* (*RdxA*); Met^r^, Cm^r^	This study
	KO 1275	*H. pylori* 26695 strain with a chloramphenicol resistance cassette in *HP1275*; Cm^r^	This study
	Com 1275	KO 1275 with *HP1275* insertion in *HP0954* (*RdxA*); Met^r^, Cm^r^	This study
Plasmid	pGEM-T	T-A cloning vector; Amp^r^	Promega, Madison, WI, USA
	pGEM-T-HP0044	pGEM-T with *HP0044* DNA fragment; Amp^r^	This study
	pGEM-T-HP0044^d^::Cm^r^	pGEM-T with *HP0044* interrupted with a chloramphenicol resistance cassette; Cm^r^	This study ^1^
	pGEM-T-RdxA_L_-P_HP1563_-HP0044-T7_ter_-RdxA_R_	pGEM-T with *HP0044* insertion in *HP0954* (*RdxA*); Met^r^, Cm^r^	This study
	pGEM-T-HP1275	pGEM-T with *HP1275* DNA fragment; Amp^r^	This study
	pGEM-T-HP1275^d^::Cm^r^	pGEM-T with *HP1275* interrupted with a chloramphenicol resistance cassette; Cm^r^	This study
	pGEM-T-RdxA_L_-P_HP1563_-HP1275-T7_ter_-RdxA_R_	pGEM-T with *HP1275* insertion in *HP0954* (*RdxA*); Met^r^, Cm^r^	This study

^1^ Amp^r^, ampicillin-resistant; Kan^r^, kanamycin-resistant; Cm^r^, chloramphenicol-resistant; and Met^r^, metronidazole-resistant. ^2^ ATCC: American Type Culture Collection.

**Table 2:** Sequences and restriction sites of the primers used in this study.

Primer	Restriction Enzyme Site	Sequence (5′→3′) ^5^
KO0044F1 ^1^	-	AATCGCTTTAATCACCGGGG
KO0044R1 ^1^	BamHI	TACAGGATCCAAAGTTTCGC
KO0044F2 ^1^	BamHI	CTTTGGATCCTGTAACCCGT
KO0044R2 ^1^	-	ATGCCATAGGCACCAGTGAT
Com0044F1 ^2^	NcoI	CCATGGCTTGATTGGAAGCACTAGCCACG
Com0044R1 ^2^	-	ATTTTTTCTTTCATATCGTAACTCCTTAAGTGTT
Com0044F2 ^2^	-	TAAGGAGTTACGATATGAAAGAAAAAATCGCTTT
Com0044R2 ^2^	KpnI	AAGGTACCTCAGTGGTGATGGTGATGATGTTCATAAAAATTCCT
KO1275F1 ^3^	-	AAACGCATGGCAAAATTTATGCG
KO1275R1 ^3^	BamHI	CCCAGGATCCTCAGGATCCG
KO1275F2 ^3^	BamHI	CTGAGGATCCTGGGAATTTC
KO1275R2 ^3^	-	GGGATATGAAATGAGATTATCCC
Com1275F1 ^4^	NcoI	CCATGGCTTGATTGGAAGCACTAGCCACG
Com1275R1 ^4^	-	ATGCTAATGTCCATATCGTAACTCCTTAAGTGTT
Com1275F2 ^4^	-	TAAGGAGTTACGATATGGACATTAGCATTTTTAG
Com1275R2 ^4^	KpnI	AAGGTACCTTAGTGGTGATGGTGATGATGAAGTTTTTCTAATAA

^1^ Primers used in the construction of the *HP0044* knockout mutant. ^2^ Primers used in the construction of the *HP0044* knockout complementary mutant. ^3^ Primers used in the construction of the *HP1275* knockout mutant. ^4^ Primers used in the construction of the *HP1275* knockout complementary mutant. ^5^ The sequences of restriction enzyme sites are underlined.

**Table 3:** Sequence comparison of the HP0044 protein and HP1275 protein from *H. pylori* wild-type strain 26695 with corresponding homologues from other Gram-negative bacteria.

	Species	UniProt AC	Identity ^1^ (%)	Similarity ^1^ (%)	Length
GDP-D-mannose dehydratase (GMD, dehydration of GDP-D-mannose)	*Helicobacter pylori* 26695	O24885	100%	100%	381 aa ^2^
	*Yersinia enterocolitica* 8081	Q56872	61.7%	83.6%	372 aa ^2^
	*Escherichia coli* K12	P0AC88	60.1%	82.7%	373 aa ^2^
	*Salmonella typhimurium* CT18	Q8Z5H1	59.6%	81.9%	373 aa ^2^
	*Vibrio cholerae* O1 biovar El Tor N16961	Q06952	61.0 %	81.8%	373 aa ^2^
Phosphomannomutase (PMM, transferring phosphate group within a mannose)	*Helicobacter pylori* 26695	O24885	100%	100%	459 aa ^2^
	*Xanthomonas campestris pv. vesicatoria*	Q3BP79	28.2%	57.4%	448 aa ^2^
	*Pseudomonas aeruginosa* PAO1	P26276	38.9%	67.2%	463 aa ^2^
	*Salmonella typhimurium* SL1344	A0A718J2B2	27.4%	55.9%	456 aa ^2^
	*Vibrio cholerae *2017V-1105**	A0A366AER7	26.4%	57.1%	454 aa ^2^
Phosphoglucomutase (PGM, transferring phosphate group within a glucose)	*Helicobacter pylori* 26695	O24885	100%	100%	459 aa ^2^
	*Xanthomonas campestris pv. vesicatoria*	Q3BP79	28.2%	57.4%	448 aa ^2^
	*Pseudomonas aeruginosa* PAO1	P26276	38.9%	67.2%	463 aa ^2^
	*Vibrio cholerae*	A0A0H5W931	21.6%	54.2%	548 aa ^2^
	*Neisseria gonorrhoeae*	P40390	40.4%	66.2%	460 aa ^2^

^1^ Data were obtained by the LALIGN program (accessed on 11 December 2021, https://fasta.bioch.virginia.edu/fasta_www2/fasta_www.cgi?rm=lalign). ^2^ aa, amino acids.

## Data Availability

The data presented in this study are available within the article.
